# Reporting to Improve Reproducibility and Facilitate Validity Assessment for Healthcare Database Studies V1.0

**DOI:** 10.1002/pds.4295

**Published:** 2017-09-15

**Authors:** Shirley V. Wang, Sebastian Schneeweiss, on behalf of the joint ISPE‐ISPOR Special Task Force on Real World Evidence in Health Care Decision Making, Marc L. Berger, Jeffrey Brown, Frank de Vries, Ian Douglas, Joshua J. Gagne, Rosa Gini, Olaf Klungel, C. Daniel Mullins, Michael D. Nguyen, Jeremy A. Rassen, Liam Smeeth, Miriam Sturkenboom

**Affiliations:** ^1^ Division of Pharmacoepidemiology and Pharmacoeconomics Brigham and Women's Hospital MA USA; ^2^ Department of Medicine Harvard Medical School MA USA; ^3^ Pfizer NY USA; ^4^ Department of Population Medicine Harvard Medical School MA USA; ^5^ Department of Clinical Pharmacy Maastricht UMC+ The Netherlands; ^6^ London School of Hygiene and Tropical Medicine England UK; ^7^ Agenzia regionale di sanità della Toscana Florence Italy; ^8^ Division of Pharmacoepidemiology & Clinical Pharmacology Utrecht University Utrecht Netherlands; ^9^ Pharmaceutical Health Services Research Department University of Maryland School of Pharmacy MA USA; ^10^ FDA Center for Drug Evaluation and Research USA; ^11^ Aetion, Inc. NY USA; ^12^ Erasmus University Medical Center Rotterdam Netherlands

**Keywords:** Transparency, reproducibility, replication, healthcare databases, pharmacoepidemiology, methods, longitudinal data

## Abstract

**Purpose:**

Defining a study population and creating an analytic dataset from longitudinal healthcare databases involves many decisions. Our objective was to catalogue scientific decisions underpinning study execution that should be reported to facilitate replication and enable assessment of validity of studies conducted in large healthcare databases.

**Methods:**

We reviewed key investigator decisions required to operate a sample of macros and software tools designed to create and analyze analytic cohorts from longitudinal streams of healthcare data. A panel of academic, regulatory, and industry experts in healthcare database analytics discussed and added to this list.

**Conclusion:**

Evidence generated from large healthcare encounter and reimbursement databases is increasingly being sought by decision‐makers. Varied terminology is used around the world for the same concepts. Agreeing on terminology and which parameters from a large catalogue are the most essential to report for replicable research would improve transparency and facilitate assessment of validity. At a minimum, reporting for a database study should provide clarity regarding operational definitions for key temporal anchors and their relation to each other when creating the analytic dataset, accompanied by an attrition table and a design diagram.

A substantial improvement in reproducibility, rigor and confidence in real world evidence generated from healthcare databases could be achieved with greater transparency about operational study parameters used to create analytic datasets from longitudinal healthcare databases.

## 1 INTRODUCTION

Modern healthcare encounter and reimbursement systems produce an abundance of electronically recorded, patient‐level longitudinal data. These data streams contain information on physician visits, hospitalizations, diagnoses made and recorded, procedures performed and billed, medications prescribed and filled, lab tests performed or results recorded, as well as many other date‐stamped items. Such temporally ordered data are used to study the effectiveness and safety of medical products, healthcare policies, and medical interventions and have become a key tool for improving the quality and affordability of healthcare.^1^, ^2^ The importance and influence of such “real world” evidence is demonstrated by commitment of governments around the world to develop infrastructure and technology to increase the capacity for use of these data in comparative effectiveness and safety research as well as health technology assessments.^3^, ^4^, ^5^, ^6^, ^7^, ^8^, ^9^, ^10^, ^11^, ^12^

Research conducted using healthcare databases currently suffers from a lack of transparency in reporting of study details.^13^, ^14^, ^15^, ^16^ This has led to high profile controversies over apparent discrepancies in results and reduced confidence in evidence generated from healthcare databases. However, subtle differences in scientific decisions regarding specific study parameters can have significant impacts on results and interpretation – as was discovered in the controversies over 3^rd^ generation oral contraceptives and risk of venous thromboembolism or statins and the risk of hip fracture.^17^, ^18^ Clarity regarding key operational decisions would have facilitated replication, assessment of validity and earlier understanding of the reasons that studies reported different findings.

The intertwined issues of transparency, reproducibility and validity cut across scientific disciplines. There has been an increasing movement towards “open science”, an umbrella term that covers study registration, data sharing, public protocols and more detailed, transparent reporting.^19^, ^20^, ^21^, ^22^, ^23^, ^24^, ^25^, ^26^, ^27^, ^28^ To address these issues in the field of healthcare database research, a Joint Task Force between the International Society for Pharmacoepidemiology (ISPE) and the International Society for Pharmacoeconomics and Outcomes Research (ISPOR) was convened to address transparency in process for database studies (e.g. “what did you plan to do?”) and transparency in study execution (e.g. “what did you actually do?). This paper led by ISPE focuses on the latter topic, reporting of the specific steps taken during study implementation to improve reproducibility and assessment of validity.

**Figure 1:**
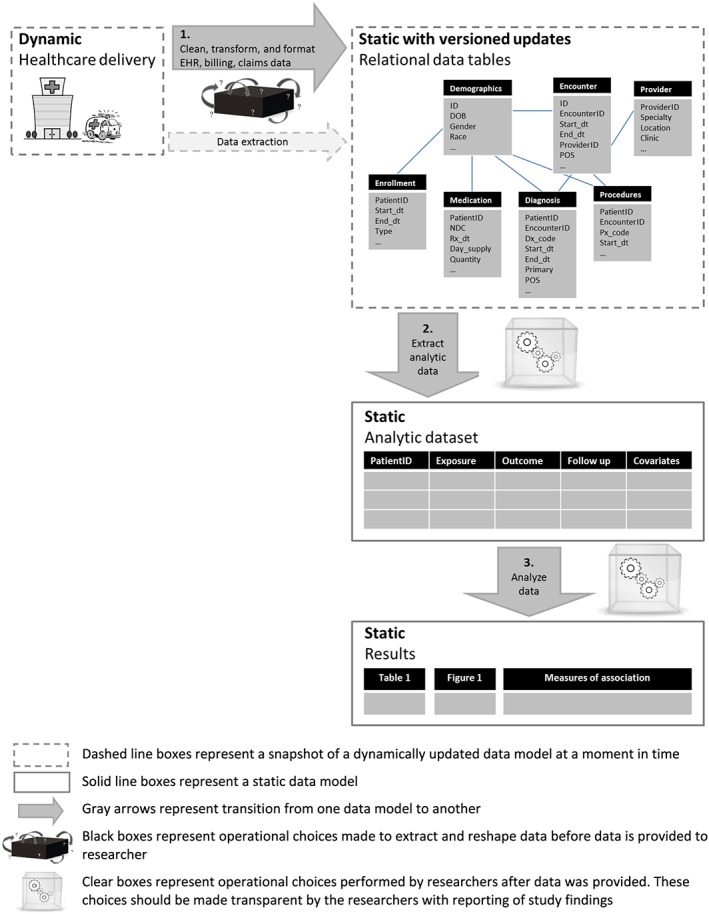
Data provenance: transitions from healthcare delivery to analysis results. [Colour figure can be viewed at wileyonlinelibrary.com]

Transparency and reproducibility in large healthcare databases is dependent on clarity regarding 1) cleaning and other pre‐processing of raw source data tables, 2) operational decisions to create an analytic dataset and 3) analytic choices (**Figure** **1**). This paper focuses on reporting of design and implementation decisions to define and create a temporally anchored study population from raw longitudinal source data (**Figure** **1** **Step 2**). A temporally anchored study population is identified by a sentinel event – an initial temporal anchor. Characteristics of patients, exposures and/or outcomes are evaluated during time periods defined in relation to the sentinel event.

However understanding how source data tables are cut, cleaned and pre‐processed prior to implementation of a research study (**Figure** **1** **Step 1**), how information is extracted from unstructured data (e.g. natural language processing of free text from clinical notes), and how the created dataset is analyzed (**Figure** **1** **Step 3**) are also important parts of reproducible research. These topics have been covered elsewhere,^14^, ^29^, ^30^, ^31^, ^32^, ^33^, ^34^, ^35^, ^36^ however we summarize key points for those data provenance steps in the online appendix.

### 1.1 Transparency

Transparency in what researchers initially intended to do protects against data dredging and cherry picking of results. It can be achieved with pre‐registration and public posting of protocols before initiation of analysis. This is addressed in detail in a companion paper led by ISPOR.^37^ Because the initially planned research and the design and methodology underlying reported results may differ, it is also important to have transparency regarding what researchers *actually did* to obtain the reported results from a healthcare database study. This can be achieved with clear reporting on the detailed operational decisions made by investigators during implementation. These decisions include how to define a study population (whom to study), and how to design and conduct an analysis (what to measure, when and how to measure it).

### 1.2 Reproducibility and replicability

Reproducibility is a characteristic of a study or a finding. A reproducible study is one for which independent investigators implementing the same methods in the same data are able to obtain the same results (direct replication^38^). In contrast, a reproducible finding is a higher order target than a reproducible study, which can be tested by conducting multiple studies that evaluate *the same question and estimand (target of inference)* but use different data and/or apply different methodology or operational decisions (conceptual replication^38^) (Table 1).

**Table 1:**
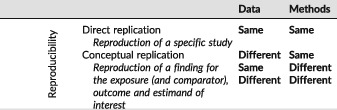
Reproducibility and replicability

Direct replicability is a necessary, but not sufficient, component of high quality research. In other words, a fully transparent and directly replicable research study is not necessarily rigorous nor does it necessarily produce valid findings. However, the transparency that makes direct replication possible means that validity of design and operational decisions can be evaluated, questioned and improved. Higher order issues such as conceptual replication of the finding can and should be evaluated as well, however, without transparency in study implementation, it can be difficult to ascertain whether superficially similar studies address the same conceptual question.

For healthcare database research, direct replication of a study means that if independent investigators applied the *same* design operational choices to the *same* longitudinal source data, they should be able to obtain the *same* results (or at least a near exact reproduction). In contrast, conceptual replication and robustness of a finding can be assessed by applying the *same* methods to *different* source data (or different years from the same source). Here, lack of replicability would not necessarily mean that one result is more “correct” than another, or refutes the results of the original. Instead, it would highlight a need for deeper inquiry to find the drivers of the differences, including differences in data definitions and quality, temporal changes or true differences in treatment effect for different populations. Conceptual replications can be further evaluated through application of *different* plausible methodologic and operational decisions to the *same* or *different* source data to evaluate how much the finding is influenced by the specific parameter combinations originally selected. This would encompass evaluation of how much reported findings vary with plausible alternative parameter choices, implementation in comparable data sources or after flawed design or operational decision is corrected. However, the scientific community cannot evaluate the validity and rigor of research methods if implementation decisions necessary for replication are not transparently reporte.

The importance of achieving consistently reproducible research is recognized in many reporting guidelines (e.g. STROBE,^34^ RECORD,^39^ PCORI Methodology Report,^40^ EnCePP^33^) and is one impetus for developing infrastructure and tools to scale up capacity for generating evidence from large healthcare database research.^3^, ^41^, ^42^, ^43^, ^44^, ^45^ Other guidelines, such as the ISPE Guidelines for Good Pharmacoepidemiology Practice (GPP) broadly cover many aspects of pharmacoepidemiology from protocol development, to responsibilities of research personnel and facilities, to human subject protection and adverse event reporting.^46^ While these guidelines certainly increase transparency, even strict adherence to existing guidance would not provide all the information necessary for full reproducibility. In recognition of this issue, ISPE formed a joint task force with ISPOR specifically focused on improving transparency, reproducibility and validity assessment for database research, and supported a complementary effort to develop a version of the RECORD reporting guidelines with a specific focus on healthcare database pharmacepidemiology.

Any replication of database research requires an exact description of the transformations performed upon the source data and how missing data are handled. Indeed, it has been demonstrated that when researchers go beyond general guidance and provide a clear report of the temporal anchors, coding algorithms, and other decisions made to create and analyze their study population(s), independent investigators following the same technical/statistical protocol and using the same data source are able to closely replicate the study population and results.^47^

### 1.3 The current status of transparency and reproducibility of healthcare database studies

Many research fields that rely on primary data collection have emphasized creation of repositories for sharing study data and analytic code.^48^, ^49^ In contrast to fields that rely on primary data collection, numerous healthcare database researchers routinely make secondary use of the same large healthcare data sources. However the legal framework that enables healthcare database researchers to license or otherwise access raw data for research often prevents public sharing both of raw source data itself as well as created analytic datasets due to patient privacy and data security concerns. Access to data and code guarantees the ability to directly replicate a study. However, the current system for multi‐user access to the same large healthcare data sources often prevents public sharing of that data. Furthermore, database studies require thousands of lines of code to create and analyze a temporally anchored study population from a large healthcare database. This is several orders of magnitude larger than the code required for analysis of a randomized trial or other dataset based on primary collection. Transparency requires clear reporting of the decisions and parameters used in study execution. While we encourage sharing data and code, we recognize that for many reasons, including data use agreements and intellectual property, this is often not possible. We emphasize that simply sharing code without extensive annotation to identify where key operational and design parameters are defined would obfuscate important scientific decisions. Clear natural language description of key operational and design details should be the basis for sharing the scientific thought process with the majority of informed consumers of evidence.

### 1.4 Recent efforts to improve transparency and reproducibility of healthcare database studies

To generate transparent and reproducible evidence that can inform decision‐making at a larger scale, many organizations have developed infrastructure to more efficiently utilize large healthcare data sources.^9^, ^50^, ^51^, ^52^, ^53^, ^54^, ^55^, ^56^ Recently developed comprehensive software tools from such organizations use different coding languages and platforms to facilitate identification of study populations, creation of temporally anchored analytic datasets, and analysis from raw longitudinal healthcare data streams. They have in common the flexibility for investigators to turn “gears and levers” at key operational touchpoints to create analytically usable, customized study populations from raw longitudinal source data tables. However, the specific parameters that must be user specified, the flexibility of the options and the underlying programming code differ. Many but not all, reusable software tools go through extensive quality checking and validation processes to provide assurance of the fidelity of the code to intended action. Transparency in quality assurance and validation processes for software tools is critically important to prevent exactly replicable findings that lack fidelity to intended design and operational parameters.

Even with tools available to facilitate creation and analysis of a temporally anchored study population from longitudinal healthcare databases, investigators must still take responsibility for publically reporting the details of their design and operational decisions. Due to the level of detail, these can be made available as online appendices or web links for publications and reports.

### 1.5 Objective

The objective of this paper was to catalogue scientific decisions made when executing a database study that are relevant for facilitating replication and assessment of validity.

We emphasize that a fully transparent study does not imply that reported parameter choices were scientifically valid; rather, the validity of a research study cannot be evaluated without transparency regarding those choices. We also note that the purpose of this paper was not to recommend specific software or suggest that studies conducted with software platforms are better than studies based on *de novo* code.

## 2 METHODS

In order to identify an initial list of key parameters that must be defined to implement a study, we reviewed 5 macro based programs and software systems designed to support healthcare database research (listed in appendix). We used this as a starting point because such programs are designed with flexible parameters to allow creation of customized study populations based on user specified scientific decisions.^54^, ^57^, ^58^, ^59^, ^60^ These flexible parameters informed our catalogue of operational decisions that would have to be transparent for an independent investigator to fully understand how a study was implemented and be able to directly replicate a study.

Our review included a convenience sample of macro based programs and software systems that were publically available, developed by or otherwise accessible to members of the Task Force. Although the software systems used a variety of coding languages, from a methodologic perspective, differences in code or coding languages are irrelevant so long as study parameters are implemented as intended by the investigator.

In our review, we identified places where an investigator had to make a scientific decision between options or create study specific inputs to create an analytic dataset from raw longitudinal source data, including details of data source, inclusion/exclusion criteria, exposure definition, outcome definition, follow up (days at risk), baseline covariates, as well as reporting on analysis methods. As we reviewed each tool, we added new parameters that had not been previously encountered and synonyms for different concepts.

After the list of parameters was compiled, the co‐authors, an international group of database experts, corresponded about these items and suggested additional parameters to include. In‐person discussions took place following the ISPE mid‐year in London (2017).

This paper was opened to comment by ISPE membership prior to publication and was endorsed by ISPE's Executive Board on July 20, 2017. The paper was also reviewed by ISPOR membership and endorsed by ISPOR leadership.

## 3 RESULTS

Our review identified many scientific decisions necessary to operate software solutions that would facilitate direct replication of an analytic cohort from raw source data captured in a longitudinal healthcare data source (Table 2). After reviewing the first two comprehensive software solutions, no parameters were added with review of additional software tools (e.g. “saturation point”). The general catalogue includes items that may not be relevant for all studies or study designs.

**Table 2:** Reporting specific parameters to increase reproducibility of database studies^*^

	Description	Example	Synonyms
**A. Reporting on data source should include:**			
A.1 Data provider	Data source name and name of organization that provided data.	Medicaid Analytic Extracts data covering 50 states from the Centers for Medicare and Medicaid Services.	
***A.2 Data extraction date (DED)***	The date (or version number) when data were extracted from the dynamic raw transactional data stream (e.g. date that the data were cut for research use by the vendor).	The source data for this research study was cut by [data vendor] on January 1st, 2017. The study included administrative claims from Jan 1st 2005 to Dec 31st 2015.	Data version, data pull
A.3 Data sampling	The search/extraction criteria applied if the source data accessible to the researcher is a subset of the data available from the vendor.		
***A.4 Source data range (SDR)***	The calendar time range of data used for the study. Note that the implemented study may use only a subset of the available data.		Study period, query period
A.5 Type of data	The domains of information available in the source data, e.g. administrative, electronic health records, inpatient versus outpatient capture, primary vs secondary care, pharmacy, lab, registry.	The administrative claims data include enrollment information, inpatient and outpatient diagnosis (ICD9/10) and procedure (ICD9/10, CPT, HCPCS) codes as well as outpatient dispensations (NDC codes) for 60 million lives covered by Insurance X. The electronic health records data include diagnosis and procedure codes from billing records, problem list entries, vital signs, prescription and laboratory orders, laboratory results, inpatient medication dispensation, as well as unstructured text found in clinical notes and reports for 100,000 patients with encounters at ABC integrated healthcare system.	
A.6 Data linkage, other supplemental data	Data linkage or supplemental data such as chart reviews or survey data not typically available with license for healthcare database.	We used Surveillance, Epidemiology, and End Results (SEER) data on prostate cancer cases from 1990 through 2013 linked to Medicare and a 5% sample of Medicare enrollees living in the same regions as the identified cases of prostate cancer over the same period of time. The linkage was created through a collaborative effort from the National Cancer Institute (NCI), and the Centers for Medicare and Medicaid Services (CMS).	
A.7 Data cleaning	Transformations to the data fields to handle missing, out of range values or logical inconsistencies. This may be at the data source level or the decisions can be made on a project specific basis.	Global cleaning: The data source was cleaned to exclude all individuals who had more than one gender reported. All dispensing claims that were missing day's supply or had 0 days’ supply were removed from the source data tables. Project specific cleaning: When calculating duration of exposure for our study population, we ignored dispensation claims that were missing or had 0 days’ supply. We used the most recently reported birth date if there was more than one birth date reported.	
A.8 Data model conversion	Format of the data, including description of decisions used to convert data to fit a Common Data Model (CDM).	The source data were converted to fit the Sentinel Common Data Model (CDM) version 5.0. Data conversion decisions can be found on our website (http://ourwebsite). Observations with missing or out of range values were not removed from the CDM tables.	
**B. Reporting on overall design should include:**			
B.1 Design diagram	A figure that contains 1st and 2nd order temporal anchors and depicts their relation to each other.	See example Figure 2.	
**C. Reporting on inclusion/exclusion criteria should include:**			
***C.1 Study entry date (SED)***	The date(s) when subjects enter the cohort.	We identified the first SED for each patient. Patients were included if all other inclusion/exclusion criteria were met at the first SED. We identified all SED for each patient. Patients entered the cohort only once, at the first SED where all other inclusion/exclusion criteria were met. We identified all SED for each patient. Patients entered the cohort at every SED where all other inclusion/exclusion criteria were met.	Index date, cohort entry date, outcome date, case date, qualifying event date, sentinel event
C.2 Person or episode level study entry	The type of entry to the cohort. For example, at the individual level (1x entry only) or at the episode level (multiple entries, each time inclusion/exclusion criteria met).		Single vs multiple entry, treatment episodes, drug eras
C.3 Sequencing of exclusions	The order in which exclusion criteria are applied, specifically whether they are applied before or after the selection of the SED(s).		Attrition table, flow diagram, CONSORT diagram
***C.4 Enrollment window (EW)***	The time window prior to SED in which an individual was required to be contributing to the data source.	Patients entered the cohort on the date of their first dispensation for Drug X or Drug Y after at least 180 days of continuous enrollment (30 day gaps allowed) without dispensings for either Drug X or Drug Y.	Observation window
C.5 Enrollment gap	The algorithm for evaluating enrollment prior to SED including whether gaps were allowed.		
C.6 Inclusion/Exclusion definition window	The time window(s) over which inclusion/exclusion criteria are defined.	Exclude from cohort if ICD‐9 codes for deep vein thrombosis (451.1x, 451.2x, 451.81, 451.9x, 453.1x, 453.2x, 453.8x, 453.9x, 453.40, 453.41, 453.42 where x represents presence of a numeric digit 0‐9 or no additional digits) were recorded in the primary diagnosis position during an inpatient stay within the 30 days prior to and including the SED. Invalid ICD‐9 codes that matched the wildcard criteria were excluded.	
C.7 Codes	The exact drug, diagnosis, procedure, lab or other codes used to define inclusion/exclusion criteria.		Concepts, vocabulary, class, domain
C.8 Frequency and temporality of codes	The temporal relation of codes in relation to each other as well as the SED. When defining temporality, be clear whether or not the SED is included in assessment windows (e.g. occurred on the same day, 2 codes for A occurred within 7 days of each other during the 30 days prior to and including the SED).		
C.9 Diagnosis position (if relevant/available)	The restrictions on codes to certain positions, e.g. primary vs. secondary. Diagnoses.		
C.10 Care setting	The restrictions on codes to those identified from certain settings, e.g. inpatient, emergency department, nursing home.		Care site, place of service, point of service, provider type
C.11 Washout for exposure	The period used to assess whether exposure at the end of the period represents new exposure.	New initiation was defined as the first dispensation for Drug X after at least 180 days without dispensation for Drug X, Y, and Z.	Lookback for exposure, event free period
C.12 Washout for outcome	The period prior to SED or ED to assess whether an outcome is incident.	Patients were excluded if they had a stroke within 180 days prior to and including the cohort entry date. Cases of stroke were excluded if there was a recorded stroke within 180 days prior.	Lookback for outcome, event free period
**D. Reporting on exposure definition should include:**			
D.1 Type of exposure	The type of exposure that is captured or measured, e.g. drug versus procedure, new use, incident, prevalent, cumulative, time‐varying.	We evaluated risk of outcome Z following incident exposure to drug X or drug Y. Incident exposure was defined as beginning on the day of the first dispensation for one of these drugs after at least 180 days without dispensations for either (SED). Patients with incident exposure to both drug X and drug Y on the same SED were excluded. The exposure risk window for patients with Drug X and Drug Y began 10 days after incident exposure and continued until 14 days past the last days supply, including refills. If a patient refilled early, the date of the early refill and subsequent refills were adjusted so that the full days supply from the initial dispensation was counted before the days supply from the next dispensation was tallied. Gaps of less than or equal to 14 days in between one dispensation plus days supply and the next dispensation for the same drug were bridged (i.e. the time was counted as continuously exposed). If patients exposed to Drug X were dispensed Drug Y or vice versa, exposure was censored. NDC codes used to define incident exposure to drug X and drug Y can be found in the appendix. Drug X was defined by NDC codes listed in the appendix. Brand and generic versions were used to define Drug X. Non pill or tablet formulations and combination pills were excluded.	
D.2 Exposure risk window (ERW)	The ERW is specific to an exposure and the outcome under investigation. For drug exposures, it is equivalent to the time between the minimum and maximum hypothesized induction time following ingestion of the molecule.		Drug era, risk window
D.2a Induction period^1^	Days on or following study entry date during which an outcome would not be counted as "exposed time" or "comparator time".		Blackout period
D.2b Stockpiling^1^	The algorithm applied to handle leftover days supply if there are early refills.		
D.2c Bridging exposure episodes^1^	The algorithm applied to handle gaps that are longer than expected if there was perfect adherence (e.g. non‐overlapping dispensation + day's supply).		Episode gap, grace period, persistence window, gap days
D.2d Exposure extension^1^	The algorithm applied to extend exposure past the days supply for the last observed dispensation in a treatment episode.		Event extension
D.3 Switching/add on	The algorithm applied to determine whether exposure should continue if another exposure begins.		Treatment episode truncation indicator
D.4 Codes, frequency and temporality of codes, diagnosis position, care setting	Description in Section C.		Concepts, vocabulary, class, domain, care site, place of service, point of service, provider type
***D.5 Exposure Assessment Window (EAW)***	A time window during which the exposure status is assessed. Exposure is defined at the end of the period. If the occurrence of exposure defines cohort entry, e.g. new initiator, then the EAW may be a point in time rather than a period. If EAW is after cohort entry, FW must begin after EAW.	We evaluated the effect of treatment intensification vs no intensification following hospitalization on disease progression. Study entry was defined by the discharge date from the hospital. The exposure assessment window started from the day after study entry and continued for 30 days. During this period, we identified whether or not treatment intensified for each patient. Intensification during this 30 day period determined exposure status during follow up. Follow up for disease progression began 31 days following study entry and continued until the firsst censoring criterion was met.	
**E. Reporting on follow‐up time should include:**			
***E.1 Follow‐up window (FW)***	The time following cohort entry during which patients are at risk to develop the outcome due to the exposure. FW is based on a biologic exposure risk window defined by minimum and maximum induction times. However, FW also accounts for censoring mechanisms.	Follow up began on the SED and continued until the earliest of discontinuation of study exposure, switching/adding comparator exposure, entry to nursing home, death, or end of study period. We included a biologically plausible induction period, therefore, follow up began 60 days after the SED and continued until the earliest of discontinuation of study exposure, switching/adding comparator exposure, entry to nursing home, death, or end of study period.	
E.2 Censoring criteria	The criteria that censor follow up.		
**F. Reporting on outcome definition should include:**			
***F.1 Event date ‐ ED***	The date of an event occurrence.	The ED was defined as the date of first inpatient admission with primary diagnosis 410.x1 after the SED and occurring within the follow up window.	Case date, measure date, observation date
F.2 Codes, frequency and temporality of codes, diagnosis position, care setting	Description in Section C.		Concepts, vocabulary, class, domain, care site, place of service, point of service, provider type
F.3. Validation	The performance characteristics of outcome algorithm if previously validated.	The outcome algorithm was validated via chart review in a population of diabetics from data source D (citation). The positive predictive value of the algorithm was 94%.	
**G. Reporting on covariate definitions should include:**			Event measures, observations
***G.1 Covariate assessment window (CW)***	The time over which patient covariates are assessed.	We assessed covariates during the 180 days prior to but not including the SED.	Baseline period
G.2 Comorbidity/risk score	The components and weights used in calculation of a risk score.	See appendix for example. Note that codes, temporality, diagnosis position and care setting should be specified for each component when applicable.	
G.3 Healthcare utilization metrics	The counts of encounters or orders over a specified time period, sometimes stratified by care setting, or type of encounter/order.	We counted the number of generics dispensed for each patient in the CAP. We counted the number of dispensations for each patient in the CAP. We counted the number of outpatient encounters recorded in the CAP. We counted the number of days with outpatient encounters recorded in the CAP. We counted the number of inpatient hospitalizations in the CAP, if admission and discharge dates for different encounters overlapped, these were "rolled up" and counted as 1 hospitalization.	
G.4 Codes, frequency and temporality of codes, diagnosis position, care setting	Description in Section C.	Baseline covariates were defined by codes from claims with service dates within 180 days prior to and including the SED. Major upper gastrointestinal bleeding was defined as inpatient hospitalization with: At least one of the following ICD‐9 diagnoses: 531.0x, 531.2x, 531.4x, 531.6x, 532.0x, 532.2x, 532.4x, 532.6x, 533.0x, 533.2x, 533.4x, 533.6x, 534.0x, 534.2x, 534.4x, 534.6x, 578.0 ‐ OR ‐ An ICD‐9 procedure code of: 44.43 ‐ OR ‐ A CPT code 43255	Concepts, vocabulary, class, domain, care site, place of service, point of service, provider type
**H. Reporting on control sampling should include:**			
H.1 Sampling strategy	The strategy applied to sample controls for identified cases (patients with ED meeting all inclusion/exclusion criteria).	We used risk set sampling without replacement to identify controls from our cohort of patients with diagnosed diabetes (inpatient or outpatient ICD‐9 diagnoses of 250.xx in any position). Up to 4 controls were randomly matched to each case on length of time since SED (in months), year of birth and gender. The random seed and sampling code can be found in the online appendix.	
H.2 Matching factors	The characteristics used to match controls to cases.		
H.3 Matching ratio	The number of controls matched to cases (fixed or variable ratio).		
**I. Reporting on statistical software should include:**			
I.1 Statistical software program used	The software package, version, settings, packages or analytic procedures.	We used: SAS 9.4 PROC LOGISTIC Cran R v3.2.1 survival package Sentinel's Routine Querying System version 2.1.1 CIDA+PSM^1^ tool Aetion Platform release 2.1.2 Cohort Safety	

Parameters in bold are key temporal anchors

The group of experts agreed that the detailed catalogue of scientific decision points that would enhance transparency and reproducibility but noted that even if every parameter were reported, there was room for different interpretation of language used to describe choices. Therefore future development of clear, shared terminology and design visualization techniques would be valuable. While sharing source data and code should be encouraged (when permissible by data use agreements and intellectual property), this would not be a sufficient substitute for transparent, natural language reporting of study parameters.

### 3.1 Data source

Researchers should specify the name of the data source, who provided the data (**A1**), the data extraction date (DED) (**A2**), data version, or data sampling strategy (**A3**) (when appropriate), as well as the years of source data used for the study (**A4**). As summarized in the appendix, source data may have subtle or profound differences depending on when the raw source data was cut for research use. Therefore, if an investigator were to run the same code to create and analyze a study population from the same data source twice, the results may not line up exactly if the investigator uses a different data version or raw longitudinal source data cut by the data holding organization at different time points.

When a researcher is granted access to only a subset of raw longitudinal source data from a data vendor, the sampling strategy and any inclusions or exclusions applied to obtain that subset should be reported. For example, one could obtain access to a 5% sample of Medicare patients flagged with diabetes in the chronic condition warehouse in the years 2010‐2014.

It is also important for researchers to describe the types of data available in the data source (**A5**) and characteristics of the data such as the median duration of person‐time within the data source. This is important for transparency and ability of decision‐makers unfamiliar with the data source to assess the validity or appropriateness of selected design choices. The data type has implications for comprehensiveness of patient data capture. For example, is the data based on administrative or electronic health records? If the latter, does the data cover only primary care, inpatient settings or an integrated health system? Does it include lab tests, results or registry data? Does it contain data on prescribed medications or dispensed medications? Is there linkage between outpatient and inpatient data? Is there linkage to other data sources? (**A6**) If so, then who did the linkage, when and how?

If the raw source data is pre‐processed, with cleaning up of messy fields or missing data, before an analytic cohort is created, the decisions in this process should be described (**A7**). For example, if the raw data is converted to a common data model (CDM) prior to creation of an analytic cohort, the CDM version should be referenced (e.g. Sentinel Common Data Model version 5.0.1,^61^ Observational Medical Outcomes Partnership Common Data Model version 5.0^62^) (**A8**). Or if individuals with inconsistent dates of birth or gender were unilaterally dropped from all relational data tables, this should be documented in meta‐data about the data source. If the data is periodically refreshed with more recent data, the date of the refresh should be reported as well as any changes in assumptions applied during the data transformation.^31^, ^32^ If cleaning decisions are made on a project specific basis rather than at a global data level, these should also be reported.

### 3.2 Design

**Figure 2:**
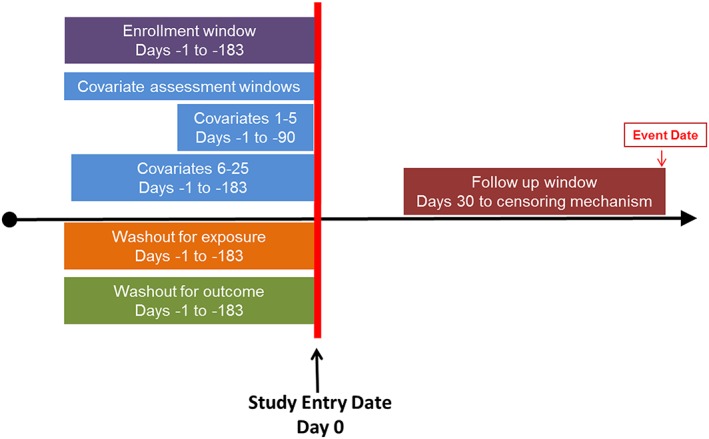
Example design diagram. [Colour figure can be viewed at wileyonlinelibrary.com]

In addition to stating the study design, researchers should provide a design diagram that provides a visual depiction of first/second order temporal anchors (**B1**, Table 3) and their relationship to each other. This diagram will provide clarity about how and when patients enter the cohort, baseline characteristics are defined as well as when follow up begins and ends. Because the terminology for similar concepts varies across research groups and software systems, visual depiction of timelines can reduce the risk of misinterpretation. We provide one example of a design diagram that depicts these temporal anchors (Figure 2). In this figure, the study entry date is day 0. A required period of enrollment is defined during the 183 days prior to but not including the study entry date. There is also washout for exposure and outcome in the 183 days prior to but not including the study entry date. There are two windows during which covariates are assessed, covariates 1‐5 are defined in the 90 days prior to but not including the study index date whereas covariates 6‐25 are defined in the 183 days prior to but not including the index date. There is an induction period following study entry so follow up for the outcome begins on day 30 and continues until a censoring mechanism is met.

**Table 3:** Key temporal anchors in design of a database study 1

Temporal Anchors	Description
**Base anchors (calendar time):**	
Data Extraction Date ‐ DED	The date when the data were extracted from the dynamic raw transactional data stream
Source Data Range ‐ SDR	The calendar time range of data used for the study. Note that the implemented study may use only a subset of the available data.
**First order anchors (event time):**	
Study Entry Date ‐ SED	The dates when subjects enter the study.
**Second order anchors (event time):**	
Enrollment Window ‐ EW	The time window prior to SED in which an individual was required to be contributing to the data source
Covariate Assessment Window ‐ CW	The time during which all patient covariates are assessed. Baseline covariate assessment should precede cohort entry in order to avoid adjusting for causal intermediates.
Follow‐Up Window ‐ FW	The time following cohort entry during which patients are at risk to develop the outcome due to the exposure.
Exposure Assessment Window ‐ EAW	The time window during which the exposure status is assessed. Exposure is defined at the end of the period. If the occurrence of exposure defines cohort entry, e.g. new initiator, then the exposure assessment may be a point in time rather than a window. If exposure assessment is after cohort entry, follow up must begin after exposure assessment.
Event Date ‐ ED	The date of an event occurrence following cohort entry
Washout for Exposure ‐ WE	The time prior to cohort entry during which there should be no exposure (or comparator).
Washout for Outcome ‐ WO	The time prior to cohort entry during which the outcome of interest should not occur

1 Anchor dates are key dates; baseline anchors identify the available source data; first order anchor dates define entry to the analytic dataset, and second order anchors are relative to the first order anchor

### 3.3 Exposure, outcome, follow up, covariates and various cohort entry criteria

A great level of detail is necessary to fully define exposure, outcome, inclusion/exclusion and covariates. As others have noted, reporting the specific codes used to define these measures is critical for transparency and reproducibility^47^, ^63^ especially in databases where there can be substantial ambiguity in code choice.

The study entry dates (**C1**) will depend on how they are selected (one entry per person versus multiple entries) (**C2**) and whether inclusion/exclusion criteria are applied before or after selection of study entry date(s) for each individual (**C3**). Reporting should include a clear description of the sequence in which criteria were applied to identify the study population, ideally in an attrition table or flow diagram, and description of whether patients were allowed to enter multiple times. If more than one exposure is evaluated, researchers should be explicit about how to handle situations where an individual meets inclusion/exclusion criteria to enter the study population as part of more than one exposure group.

Also critical are other key investigator decisions including 1) criteria for ensuring that healthcare encounters would be captured in the data (e.g. continuous enrollment for a period of time, with or without allowable gaps) (**C4, C5**), 2) specific codes used, the frequency and temporality of codes in relation to each other and the study entry date (**C6‐C8**), 3) diagnosis position (**C9**) and care settings (**C10**) (e.g. primary diagnosis in an inpatient setting). Whenever defining temporal anchors, whether or not time windows are inclusive of the study entry date should be articulated. Some studies use multiple coding systems when defining parameters. For example, studies that span the transition from ICD‐9 to ICD 10 in the United States or studies that involve data from multiple countries or delivery systems. If coding algorithms are mapped from one coding system to another, details about how the codes were mapped should be reported.

When “wildcards” are used to summarize code lists instead of listing out every single potential code, the definition of the wildcard should be specified. For example, if someone uses “x” as a wildcard in an algorithm to define a baseline covariate (e.g. ICD‐9 codes 410.x1), the definition should indicate over what time period in relation to study entry (covariate assessment window – CW), which care settings to look in (C11), whether to include only primary diagnoses (C10), and whether the wildcard “x” includes only digits 0‐9 or also includes the case of no additional digits recorded. Furthermore, when wildcards are used, it should be clear whether invalid codes found with a wildcard match in the relevant digit were excluded (e.g. 410.&1 is not a valid code but matches 410.x1).

It is important to report on who can be included in a study. Reporting should include specification of what type of exposure measurement is under investigation, for example prevalent versus incident exposure (**D1**).^64^ If the latter, the criteria used to define incidence, including the washout window, should be clearly specified (**C11**). For example, incidence with respect to the exposure of interest only, the entire drug class, exposure and comparator, etc. When relevant, place of service used to define exposure should also be specified (e.g. inpatient versus outpatient).

Type of exposure (**D1**), when exposure is assessed and duration of exposure influence who is selected into the study and how long they are followed. When defining drug exposures, investigators make decisions regarding the intended length of prescriptions as well as hypothesized duration of exposure effect. Operationally, these definitions may involve induction periods, algorithms for stockpiling of re‐filled drugs, creating treatment episodes by allowing gaps in exposure of up to X days to be bridged, extending the risk window beyond the end of days’ supply or other algorithms (**D2, D3**). The purpose of applying such algorithms to the data captured in healthcare databases is to more accurately measure the hypothesized biologic exposure risk window (ERW). The ERW is specific to an exposure and the outcome under investigation. For drug exposures, it is equivalent to the difference between the minimum and maximum induction time following ingestion of a molecule.^65^, ^66^ Similar decisions are necessary to define timing and duration of hypothesized biologic effect for non‐drug exposures. These decisions are necessary to define days at risk while exposed and should be explicitly stated. There may be data missing for elements such as days’ supply or number of tablets. Decisions about how to handle missingness should be articulated. When describing the study population, reporting on the average starting or daily dose can facilitate understanding of variation in findings between similar studies conducted in different databases where dosing patterns may differ. Specific codes, formulations, temporality, diagnosis position and care settings should be reported when relevant (**D4**).

For some studies, exposure is assessed after study entry (**D5**). For example, a study evaluating the effect of treatment intensification versus no intensification on disease progression after a hospitalization could define study entry as the date of discharge and follow up for outcomes after an exposure assessment window (EAW) during which treatment intensification status is defined. The ERW and follow up for an outcome should not begin until after EAW has concluded.^67^ The timing of EAW relative to study entry and follow up should be clearly reported when relevant.

The analytic follow up window (FW) covers the interval during which outcome occurrence could be influenced by exposure (**E1**). The analytic follow up is based on the biologic exposure risk, but the actual time at risk included may also be defined by censoring mechanisms. These censoring mechanisms should be enumerated in time to event analyses (**E2**). Reasons for censoring may include events such as occurrence of the outcome of interest, end of exposure, death, disenrollment, switching/adding medication, entering a nursing home, or use of a fixed follow‐up window (e.g. intention to treat).

Outcome surveillance decisions can strongly affect study results. In defining the outcome of interest, investigators should specify whether a washout period prior to the study entry date was applied to capture incident events (**C12**). If a washout period was applied, it should be clear whether the washout included or excluded the study entry date. The timing of the event date (**F1**) relative to the specific codes used and restrictions to certain care settings or diagnosis position should be reported if they are part of the outcome definition (**F2**). If the algorithm used to define the outcome was previously validated, a citation and performance characteristics such as positive predictive value should be reported (**F3**).

The same considerations outlined above for outcome definition apply to covariates (**G1, G4**). If a comorbidity score is defined for the study population, there should be a clear description of the score components, when and how they were measured, and the weights applied (**G2**, Appendix C). Citations often link to papers which evaluate multiple versions of a score, and it can be unclear which one was applied in the study. When medical utilization metrics are reported, there should be details about how each metric is calculated as part of the report (**G3**). For example, in counts of medical utilization, one must be clear if counts of healthcare visits are unique by day or unique by encounter identifier and whether they include all encounters or only those from specific places of service. Hospitalizations are sometimes “rolled up” and counted only once if the admission and discharge dates are contiguous or overlapping. Patients may have encounters in multiple care settings on the same date. All encounters may be counted or an algorithm applied to determine which ones are included in utilization metrics. Different investigator choices will result in different counts.

If sampling controls for a case‐control study, how and when controls are sampled should be clearly specified. Reporting should include the sampling strategy (**H1**), whether it is base case, risk set or survivor sampling. If matching factors are used, these should be listed and the algorithms for defining them made available (**H2**). The number and ratio of controls should be reported, including whether the ratio is fixed or variable and whether sampling is with or without replacement (**H3**). If multiple potential matches are available, the decision rules for which to select should be stated.

In addition, the statistical software program or platform used to create the study population and run the analysis should be detailed, including specific software version, settings, procedures or packages (**I1**).

The catalogue of items in **Table** 2 are important to report in detail in order to achieve transparent scientific decisions defining study populations and replicable creation of analytic datasets from longitudinal healthcare databases. We have highlighted in **Table** 3 key temporal anchors that are essential to report in the methods section of a paper, ideally accompanied with a design diagram (**Figure** **2**). Other items from **Table** 2 should be included with peer reviewed papers or other public reports, but may be reported in online appendices or as referenced web pages.

After creating an analytic dataset from raw longitudinal data streams, there are numerous potential ways to analyze a created analytic dataset and address confounding. Some of the most common methods used in healthcare database research include multivariable regression and summary score methods (propensity score or disease risk score matching, weighting, stratification).^68^, ^69^ Other methods include instrumental variable analysis, standardization and stratification. Each of these methods comes with their own set of assumptions and details of implementation which must be reported to assess adequacy of those methods and obtain reproducible results. In the appendix, we highlight important descriptive or comparative results to report for several commonly used analytic methods (Appendix D).

## 4 DISCUSSION

Evidence generated from large healthcare databases is increasingly being sought by decision‐makers around the world. However, publication of database study results is often accompanied by study design reported at a highly conceptual level, without enough information for readers to understand the temporality of how patients entered the study, or how exposure, outcome and covariates were operationally defined in relation to study entry. Only after decision‐makers and peer‐reviewers are reasonably confident that they know the actual steps implemented by the original researchers can they assess whether or not they agree with the validity of those choices or evaluate the reproducibility and rigor of the original study findings.

Stakeholders involved in healthcare are increasingly interested in evaluating additional streams of evidence beyond randomized clinical trials and are turning their attention toward real‐world evidence from large healthcare database studies. This interest has led to groundbreaking infrastructure and software to scale up capacity to generate database evidence from public and private stakeholders. The United States FDA's Sentinel System is one example of a large scale effort to create an open source analytic infrastructure. Supported by FDA to achieve its public health surveillance mission, the tools and infrastructure are also available to the research community through Reagan Udall Foundation's IMEDS system. Sentinel has committed itself to transparency through online posting of study protocols, final reports, and study specifications, including temporal anchors, how data are processed into a common data model, and study design details. Similarly, the Canadian government, the European Medicines Agency (EMA) and several countries in Asia have developed consortia to facilitate transparent evidence generation from healthcare databases, including the Canadian Network for Observational Drug Effect Studies (CNODES),^8^ Innovative Medicines Initiative (IMI), ENCePP^70^ and others.^9^

These efforts have made great strides in improving capacity for transparent evidence generation from large healthcare databases, however many involve closed systems that do not influence research conducted outside of the respective networks. Currently, there is not a clear roadmap for how the field should proceed. This is reflected in policies around the world. In the US, the recently passed 21^st^ Century Cures Act and Prescription Drug User Fee Act (PDUFA VI) include sections on evaluating when and how to make greater use of real world evidence to support regulatory decisions. In the EU, there is exploration of adaptive pathways to bring drugs to market more quickly by using healthcare database evidence to make approval decisions^11^ and active work on harmonizing policies on use of real ‐world evidence from databases to inform health technology assessment decisions.^12^

Regardless of whether a study is conducted with software tools or *de novo* code, as part of a network or independently, a substantial improvement in transparency of design and implementation of healthcare database research could be achieved if specific design and operation decisions were routinely reported. We encourage researchers to prepare appendices that report in detail 1) data source provenance including data extraction date or version and years covered, 2) key temporal anchors (ideally with a design diagram), 3) detailed algorithms to define patient characteristics, inclusion or exclusion criteria, and 4) attrition table with baseline characteristics of the study population before applying methods to deal with confounding. The ultimate measure of transparency is whether a study could be directly replicated by a qualified independent investigator based on publically reported information. While sharing data and code should be encouraged whenever data use agreements and intellectual property permit, in many cases this is not possible. Even if data and code are shared, clear, natural language description would be necessary for transparency and the ability to evaluate the validity of scientific decisions.

In many cases, attempts from an independent investigator to directly replicate a study will be hampered by data use agreements that prohibit public sharing of source data tables and differences in source data tables accessed from the same data holder at different times. Nevertheless, understanding how closely findings can be replicated by an independent investigator when using the same data source over the same time period would be valuable and informative. Similarly, evaluation of variation in findings from attempts to conceptually replicate an original study using different source data or plausible alternative parameter choices can provide substantial insights. Our ability to understand observed differences in findings after either direct or conceptual replication relies on clarity and transparency of the scientific decisions originally implemented.

This paper provides a catalogue of specific items to report to improve reproducibility and facilitate assessment of validity of healthcare database analyses. We expect that it will grow and change over time with input from additional stakeholders. This catalogue could be used to support parallel efforts to improve transparency and reproducibility of evidence from database research. For example, we noted that the terminology used by different research groups to describe similar concepts varied. A next step could include development of shared terminology and structured reporting templates. We also had consensus within our task force that a limited number of parameters are absolutely necessary to recreate a study population, however there was disagreement on which. Empirical evaluation of the frequency and impact of lack of transparency on the catalogue of specific operational parameters on replicability of published database studies would be a valuable next step. Empirical data could inform future policies and guidelines for reporting on database studies for journals, regulators, health technology assessment bodies and other healthcare decision‐makers, where greater priority could be placed on reporting specific parameters with high demonstrated influence on replicability. It could also help stakeholders create policies that triage limited resources by focusing on database evidence where reporting is transparent enough that validity and relevance of scientific choices can be assessed. By aligning incentives of major stakeholders, the conduct and reporting of database research will change for the better. This will increase the confidence of decision‐makers in real‐world evidence from large healthcare databases.

## ETHICS STATEMENT

The authors state that no ethical approval was needed.

## Supporting information

Appendix A. Data preparation and pre‐processing
^†^. Reviewed software tools
Appendix C. Comorbidity score example
Appendix D. Describing and analyzing database studies

## Contributors to the joint ISPE‐ISPOR Special Task Force on Real World Evidence in Health Care Decision Making

### Co‐Lead

Shirley V. Wang PhD, ScM: Division of Pharmacoepidemiology and Pharmacoeconomics, Brigham and Women's Hospital Department of Medicine, Harvard Medical School
Sebastian Schneeweiss MD, ScD: Division of Pharmacoepidemiology and Pharmacoeconomics, Brigham and Women's Hospital, Department of Medicine, Harvard Medical School

### Writing group

Marc L. Berger MD: Pfizer
Jeffrey Brown PhD: Department of Population Medicine, Harvard Medical School
Frank de Vries PharmD, PhD: Dept. Clinical Pharmacy, Maastricht UMC+, The Netherlands
Ian Douglas PhD: London School of Hygiene and Tropical Medicine
Joshua J. Gagne PharmD, ScD: Division of Pharmacoepidemiology and Pharmacoeconomics, Brigham and Women's Hospital, Department of Medicine, Harvard Medical School
Rosa Gini PhD, MSc: Agenzia regionale di sanità della Toscana, Florence, Italy
Olaf Klungel PhD: Division of Pharmacoepidemiology & Clinical Pharmacology, Utrecht University
C. Daniel Mullins PhD: Pharmaceutical Health Services Research Department, University of Maryland School of Pharmacy
Michael D. Nguyen MD: FDA Center for Drug Evaluation and Research
Jeremy A. Rassen ScD: Aetion, Inc.
Liam Smeeth MSc, PhD: London School of Hygiene and Tropical Medicine
Miriam Sturkenboom PhD, MSc: Erasmus University Medical Center Rotterdam

## Participated in small group discussion and/or provided substantial feedback prior to ISPE/ISPOR membership review

Andrew Bate PhD: Pfizer
Alison Bourke MSC, MRPharm.S: QuintilesIMS
Suzanne M. Cadarette PhD: Leslie Dan Faculty of Pharmacy, University of Toronto
Tobias Gerhard BSPharm, PhD: Department of Pharmacy Practice and Administration and Institute for Health, Health Care Policy and Aging Research
Robert Glynn ScD: Division of Pharmacoepidemiology, Brigham & Women's Hospital, Department of Medicine, Harvard Medical School
Krista Huybrechts MS, PhD: Division of Pharmacoepidemiology and Pharmacoeconomics, Brigham and Women's Hospital, Department of Medicine, Harvard Medical School
Kiyoshi Kubota MD, PhD: NPO Drug Safety Research Unit Japan
Amr Makady: Zorginstituut Nederland
Fredrik Nyberg MD, PhD: AstraZeneca
Mary E Ritchey PhD: RTI Health Solutions
Ken Rothman DrPH: RTI Health Solutions
Sengwee Toh ScD: Department of Population Medicine, Harvard Medical School
