# Supplementary material for: Reporting to Improve Reproducibility and Facilitate Validity Assessment for Healthcare Database Studies V1.0
Source: Pharmacoepidemiol Drug Saf. 2017 Sep 15;26(9):1018–32. doi: 10.1002/pds.4295 (PMC5639362; doi:10.1002/pds.4295)
Supplement: Supplementary file 1 — Appendix A. Data preparation and pre‐processing †. Reviewed software tools Appendix C. Comorbidity score example Appendix D. Describing and analyzing database studies [file PDS-26-1018-s001.docx]

**Appendix A. Data preparation and pre-processing**

*Data cut*

Although accessing secondary healthcare databases for the same time period from the same database should in theory provide the same source data for independent investigators, in practice, there may be subtle differences in raw longitudinal data. The timing of the data cut from a healthcare data source can impact study findings.[^1^](#_ENREF_1)^,^[^2^](#_ENREF_2) Some have shifts in populations whose data are eligible for research use over time. For example, electronic health record based primary care research databases in the United Kingdom have shifts in participation over time.[^3^](#_ENREF_3)^,^[^4^](#_ENREF_4) When a new organization contributes to a healthcare database covers, it could come with years of historical patient data that would be included in new data cuts for researchers, but not be present in old data cuts over the same time frame from the same vendor. Conversely, the population whose data is eligible for research could shrink over time, in which case there would be less patient data available in newer cuts of data. As another example, there could be shifts in which populations in a data source are considered “administrative services only” over time and therefore have variable inclusion in data cuts provided for research purposes. There may also be shifts in source data pending final adjudication of claims or due to back dated entries in the EHR.

*Data cleaning and transformation*

Research groups often make study-specific decisions when cleaning source data prior to use for research studies.[^1^](#_ENREF_1)^,^[^2^](#_ENREF_2) This may be at the global level or project specific. Data cleaning decisions such as whether to drop patients without a recorded date of birth or inconsistent gender recorded, whether to adjust (seemingly) out of range values (e.g. cost = <$0, BMI = 150, day’s supply dispensed= 300) can be applied globally so that all projects using the data are affected or on a project specific basis so other researchers using the same source data may make different choices. Regardless of where these data cleaning decisions are made, they should be documented.

*Data model*

There have been massive efforts to develop Common Data Models (CDM) to facilitate standardized analyses across healthcare databases from organizations such as the Food and Drug Administration’s Sentinel Program, the Observational Medical Outcomes Partnership, and the Patient-Centered Outcomes research network (PCORnet) among others.[^5-7^](#_ENREF_5) Use of CDMs involves conversion of source data to fit standardized data structures, including relational tables with specific variables and variable formats. Some granularity of data may be lost during conversion; decision rules about how to convert may vary depending on who is doing the conversion and which CDM they are converting to.

Conversion to a CDM is an example of a global data “cleaning” transformation. If source data tables are converted to a CDM, the CDM version should be cited and decisions about how to convert source data tables into CDM data tables should be documented in meta-data along with date of data cut, data version or other details of data pull methodology. Many organizations apply their own algorithms when converting from their source tables to a CDM. Meta-data about global cleaning or transformation decisions should be available to researchers using the transformed data.

Data cleaning decisions, timing of data cuts, and use of different CDMs could have subtle or profound impact on results.

**Appendix B. Reviewed software tools**

We reviewed a convenience sample of software tools:

1. United States Food and Drug Administration (FDA) Sentinel’s Cohort Identification and Descriptive Analysis (CIDA) + Propensity Score Matching tool version 3.3.0 (<https://www.sentinelinitiative.org/sentinel/surveillance-tools/routine-querying-tools/routine-querying-system>)
2. Aetion, Inc.’s Comparative Effectiveness and Safety platforms version 2.2 (<https://www.aetion.com/>)
3. Observational Health Data Sciences and Informatics Atlas version 2.0.0 (<https://ohdsi.org/analytic-tools/>)
4. Observational Health Data Sciences and Informatics CohortMethod version 2.2.2 (<https://github.com/OHDSI/CohortMethod>)
5. Innovation in Medical Evidence in Development and Surveillance (IMEDs) Regularized Identification of Cohorts (RICO) version 1.2 (<http://imeds.reaganudall.org/RICO>)

**Appendix C. Comorbidity score example**

****************************************************************************************;

* Program: combined_comorbidity_score_code.sas ;

* ;

* Purpose: To compute combined comorbidity scores based on ICD-9 codes ;

* ;

* Description: This code takes in a list of ICD-9 codes for ;

* ;

* Citation: Gagne JJ, Glynn RJ, Avorn J, Levin R, Schneeweiss S. A combined ;

* comorbidity score predicted mortality in elderly patients better ;

* than existing scores. Journal of Clinical Epidemiology 2011 Jan ;

* 3 [Epub ahead of print] ;

* ;

* Definitions: datain = input dataset containing diagnosis codes recorded within

* the covariate assessment window ; ;

* ICD9variable = ICD-9 codes ;

* ;

* Comments: After merging the final data file (combinedcomorbidityscore) with ;

* the analytic data file, patients with missing combined comorbidity ;

* score values (i.e. combinedscore = .) should be set to 0 as these ;

* patients did not have any of the component conditions. ;

* ;

****************************************************************************************;

data conditions;

set datain(rename=(ICD9variable = ICD));

length disease $25;

disease = 'nopoints';

if substr(ICD,1,3) = '196' or substr(ICD,1,3) = '197' or

substr(ICD,1,3) = '198' or substr(ICD,1,3) = '199'

then disease = 'metastatic_romano';

if ICD = '40201' or ICD = '40211' or ICD = '40291' or

substr(ICD,1,4) = '4293' or substr(ICD,1,3) = '425' or

substr(ICD,1,3) = '428' then disease = 'chf_romano';

if substr(ICD,1,4) = '3310' or substr(ICD,1,4) = '3311' or

substr(ICD,1,4) = '3312' or substr(ICD,1,3) = '290'

then disease = 'dementia_romano';

if ICD = '40311' or ICD = '40391' or ICD = '40412' or

ICD = '40492' or substr(ICD,1,3) = '585' or

substr(ICD,1,3) = '586' or substr(ICD,1,4) = 'V420' or

substr(ICD,1,4) = 'V451' or substr(ICD,1,4) = 'V560' or

substr(ICD,1,4) = 'V568' then disease = 'renal_elixhauser';

if '260' <= substr(ICD,1,3) <= '263'

then disease = 'wtloss_elixhauser';

if substr(ICD,1,3) = '342' or substr(ICD,1,3) = '344'

then disease = 'hemiplegia_romano';

if substr(ICD,1,4) = '2911' or substr(ICD,1,4) = '2912' or

substr(ICD,1,4) = '2915' or substr(ICD,1,4) = '2918' or

substr(ICD,1,4) = '2919' or

'30390' <= ICD <= '30393' or

'30500' <= ICD <= '30503' or substr(ICD,1,4) = 'V113'

then disease = 'alcohol_elixhauser';

if '140' <= substr(ICD,1,3) <= '171' or

'174' <= substr(ICD,1,3) <= '195' or

substr(ICD,1,4) = '2730' or

substr(ICD,1,4) = '2733' or

substr(ICD,1,5) = 'V1046' or

'200' <= substr(ICD,1,3) <= '208'

then disease = 'tumor_romano';

if ICD = '42610' or ICD = '42611' or ICD = '42613' or

'4262' <= substr(ICD,1,4) <= '4264' or

'42650' <= ICD <= '42653' or

'4266' <= substr(ICD,1,4) <= '4268' or

substr(ICD,1,4) = '4270' or substr(ICD,1,4) = '4272' or

ICD = '42731' or ICD = '42760' or substr(ICD,1,4) = '4279' or

substr(ICD,1,4) = '7850' or substr(ICD,1,4) = 'V450' or

substr(ICD,1,4) = 'V533'

then disease = 'arrhythmia_elixhauser';

if substr(ICD,1,4) = '4150' or substr(ICD,1,4) = '4168' or

substr(ICD,1,4) = '4169' or substr(ICD,1,3) = '491' or

substr(ICD,1,3) = '492' or substr(ICD,1,3) = '493' or

substr(ICD,1,3) = '494' or substr(ICD,1,3) = '496'

then disease = 'pulmonarydz_romano';

if '2860' <= substr(ICD,1,4) <= '2869' or

substr(ICD,1,4) = '2871' or

'2873' <= substr(ICD,1,4) <= '2875'

then disease = 'coagulopathy_elixhauser';

if '25040' <= ICD <= '25073' or

'25090' <= ICD <= '25093' then disease = 'compdiabetes_elixhauser';

if '2801' <= substr(ICD,1,4) <= '2819' or

substr(ICD,1,4) = '2859' then disease = 'anemia_elixhauser';

if '2760' <= substr(ICD,1,4) <= '2769'

then disease = 'electrolytes_elixhauser';

if ICD = '07032' or ICD = '07033' or ICD = '07054' or

substr(ICD,1,4) = '4560' or substr(ICD,1,4) = '4561' or

ICD = '45620' or ICD = '45621' or

substr(ICD,1,4) = '5710' or substr(ICD,1,4) = '5712' or

substr(ICD,1,4) = '5713' or

'57140' <= ICD <= '57149' or substr(ICD,1,4) = '5715' or

substr(ICD,1,4) = '5716' or substr(ICD,1,4) = '5718' or

substr(ICD,1,4) = '5719' or substr(ICD,1,4) = '5723' or

substr(ICD,1,4) = '5728' or substr(ICD,1,4) = 'V427'

then disease = 'liver_elixhauser';

if '4400' <= substr(ICD,1,4) <= '4409' or

substr(ICD,1,4) = '4412' or substr(ICD,1,4) = '4414' or

substr(ICD,1,4) = '4417' or substr(ICD,1,4) = '4419' or

'4431' <= substr(ICD,1,4) <= '4439' or

substr(ICD,1,4) = '4471' or substr(ICD,1,4) = '5571' or

substr(ICD,1,4) = '5579' or substr(ICD,1,4) = 'V434'

then disease = 'pvd_elixhauser';

if '29500' <= ICD <= '29899' or

ICD = '29910' or ICD = '29911'

then disease = 'psychosis_elixhauser';

if substr(ICD,1,3) = '416' or substr(ICD,1,4) = '4179'

then disease = 'pulmcirc_elixhauser';

if substr(ICD,1,3) = '042' or substr(ICD,1,3) = '043' or

substr(ICD,1,3) = '044' then disease = 'hivaids_romano';

if substr(ICD,1,4) = '4011' or substr(ICD,1,4) = '4019' or

ICD = '40210' or ICD = '40290' or ICD = '40410' or ICD = '40490' or

ICD = '40511' or ICD = '40519' or ICD = '40591' or ICD = '40599'

then disease = 'hypertension_elixhauser';

if disease ^= 'nopoints';

run;

proc sort nodupkey data = conditions;

by uniqueidentifier disease;

run;

*Applying the weights;

data conditionweights;

set conditions;

weight = 0;

if disease = 'metastatic_romano' then weight = 5;

if disease = 'chf_romano' then weight = 2;

if disease = 'dementia_romano' then weight = 2;

if disease = 'renal_elixhauser' then weight = 2;

if disease = 'wtloss_elixhauser' then weight = 2;

if disease = 'hemiplegia_romano' then weight = 1;

if disease = 'alcohol_elixhauser' then weight = 1;

if disease = 'tumor_romano' then weight = 1;

if disease = 'arrhythmia_elixhauser' then weight = 1;

if disease = 'pulmonarydz_romano' then weight = 1;

if disease = 'coagulopathy_elixhauser' then weight = 1;

if disease = 'compdiabetes_elixhauser' then weight = 1;

if disease = 'anemia_elixhauser' then weight = 1;

if disease = 'electrolytes_elixhauser' then weight = 1;

if disease = 'liver_elixhauser' then weight = 1;

if disease = 'pvd_elixhauser' then weight = 1;

if disease = 'psychosis_elixhauser' then weight = 1;

if disease = 'pulmcirc_elixhauser' then weight = 1;

if disease = 'hivaids_romano' then weight = -1;

if disease = 'hypertension_elixhauser' then weight = -1;

keep uniqueidentifier disease weight;

run;

*Summing the weights;

data combinedcomorbidityscore;

set conditionweights(keep = uniqueidentifier weight);

by uniqueidentifier;

if first.uniqueidentifier then combinedscore = 0;

combinedscore + weight;

if last.uniqueidentifier then output;

keep uniqueidentifier combinedscore;

run;

*Note: patients not included in the final data set (combinedcomorbidityscore) did not

have any of the component conditions. Therefore, be sure to set their combined comorbidity

score values to zero;

| **Appendix D. Describing and analyzing database studies** | |
| --- | --- |
|  | **Description** |
| **A. Reporting of descriptive results should include:** | |
| Flow diagram/attrition table | Including items such as: |
|  | Inclusion and exclusion criteria in the sequence they were applied to the data |
|  | Number of patients after application of each criterion |
| Describing patient characteristics of overall population | Including items such as: |
|  | Number of patients |
|  | N/% or mean (sd) of baseline characteristics |
| Describing outcomes and follow up in overall population | Including items such as: |
|  | Person-years of follow-up |
|  | Mean, median follow-up time |
|  | Reasons for censoring with numbers of subjects censored |
|  | Number of health outcomes of interest (HOI) |
|  | Risk per 1,000 persons |
|  | Rate per 1,000 person-years |
|  |  |
| **B. Reporting of comparative results should include:** | |
| Comparing patient characteristics for each exposure group | Including items such as: |
|  | Number of patients |
|  | N/% or mean (sd) of patient characteristics |
|  | Absolute or standardized differences for compared groups |
|  | Mahalanobis distance |
| Describing outcomes and follow up for each exposure group | Including items such as: |
|  | Person-years of follow up |
|  | Mean, median follow-up time |
|  | Reasons for censoring with numbers of subjects censored |
|  | Number of health outcomes of interest (HOI) |
|  | Risk per 1,000 persons |
|  | Rate per 1,000 person-years |
| Relative measure of association (ratio) | Including items such as: |
|  | Unadjusted and adjusted results |
|  | Pre-specified subgroup analyses |
| Absolute measure of association (difference) | Including items such as: |
|  | Unadjusted and adjusted |
|  | Pre-specified subgroup analyses |
| Additional diagnostic results when propensity score is used | Including items such as: |
|  | Figure with propensity score distribution pre and post matching |
|  | Tables for unmatched and matched population characteristics |
|  | Tables for stratified population characteristics |
|  | Tables for unweighted and weighted population characteristics |
|  | Mean and distribution of weights |
|  | N/% contributing to matched, trimmed, truncated or weighted analyses |
| Additional diagnostic results when instrumental variable analysis is used | Table with distribution of population characteristics across levels of instrument |
|  | Table with distribution of outcomes across levels of instruments |
|  | Strength of association between instrument and exposure |
|  | (e.g. odds ratio, risk difference, partial R²) |
|  | Results of falsification tests: |
|  | assumption that instrument does not affect outcome except through treatment |
|  | assumption that instrument and outcome do not have common causes |
|  |  |
| **C. Reporting of risk-adjustment methods should include:** | |
| Estimand | What is being estimated with the risk-adjusted analytic method? |
|  | (e.g. average effect among treated (ATT), average treatment effect (ATE), |
|  | marginal vs. conditional effect) |
| Measures of variability due to chance | How are standard errors obtained? (e.g. model-based, bootstrap, robust) |
| Methods used for confounder adjustment: |  |
| *Direct or indirect standardization* | What is the standard (reference) population? |
|  | What covariates are used for standardization? |
| *Stratification (on 1 or more covariates)* | Which covariates define strata? |
| *Multivariable outcome regression model* | What kind of model was used? (e.g. survival, binary, Poisson) |
|  | Which covariates were used and how did they enter the model? |
|  | (e.g. binary, categorical) |
| *Propensity score model* | What kind of model was used? (e.g. logistic, multinomial) |
|  | Which covariates were used and how did they enter the model? |
|  | (e.g. binary, categorical) |
| *If PS-Matching* | What matching algorithm, what caliper and on what scale? |
|  | (e.g. 0.025 standard deviations on the probability scale) |
|  | What matching ratio? (e.g. fixed 1:1, variable 1:5) |
| *If PS-Stratification* | How are strata defined? |
|  | (e.g. deciles, centiles calculated among the exposed) |
|  | Is trimming implemented before or after strata definition? |
| *If PS-Weighting* | How are the weights calculated? |
|  | Are the weights trimmed, truncated or stabilized? |
| *Instrumental variable analysis* | What kind of model was used (e.g. 2 stage least squares) |
| *Matching* | If the design involved matching, how did the analysis account for matching factors? |

**1.** Brown JS, Kahn M, Toh S. Data quality assessment for comparative effectiveness research in distributed data networks. *Medical care.* Aug 2013;51(8 Suppl 3):S22-29.

**2.** Kahn MG, Brown JS, Chun AT, et al. Transparent reporting of data quality in distributed data networks. *Egems.* 2015;3(1):1052.

**3.** Herrett E, Gallagher AM, Bhaskaran K, et al. Data Resource Profile: Clinical Practice Research Datalink (CPRD). *International journal of epidemiology.* June 6, 2015 2015.

**4.** Team THINTR. THIN Database. 2015.

**5.** OMOP Common Data Model. <http://omop.org/CDM>. Accessed 3/21/2016.

**6.** Mini-Sentinel I. *Mini-Sentinel: Overview and Description of the Common Data Model v5.0.1.*

**7.** Network. PTNP-CCR. PCORnet Common Data Model. 2016; <http://www.pcornet.org/pcornet-common-data-model/>.
